# Comparison of Machine Learning Methods towards Developing Interpretable Polyamide Property Prediction

**DOI:** 10.3390/polym13213653

**Published:** 2021-10-23

**Authors:** Franklin Langlang Lee, Jaehong Park, Sushmit Goyal, Yousef Qaroush, Shihu Wang, Hong Yoon, Aravind Rammohan, Youngseon Shim

**Affiliations:** 1 Science and Technology Division, Corning Incorporated, Corning, NY 14831, USA; GoyalS@corning.com (S.G.); QaroushYK@corning.com (Y.Q.); WangS7@Corning.com (S.W.); RammohanA@corning.com (A.R.); 2 CSE Team, Data & Information Technology (DIT) Center, Samsung Electronics Co, Ltd., Samsungjeonja-ro, Hwaseong 18448, Korea; jhong14.park@samsung.com (J.P.); ys1231.shim@samsung.com (Y.S.); 3 Corning Technology Center Korea, Corning Precision Materials Co., Ltd., 212 Tangjeong-ro, Asan 31454, Korea; HongYoon@corning.com

**Keywords:** machine learning 1, polyamide 2, QSPR 3

## Abstract

Polyamides are often used for their superior thermal, mechanical, and chemical properties. They form a diverse set of materials that have a large variation in properties between linear to aromatic compounds, which renders the traditional quantitative structure–property relationship (QSPR) challenging. We use extended connectivity fingerprints (ECFP) and traditional QSPR fingerprints to develop machine learning models to perform high fidelity prediction of glass transition temperature ($T_{g}$), melting temperature ($T_{m}$), density (ρ), and tensile modulus (E). The non-linear model using random forest is in general found to be more accurate than linear regression; however, using feature selection or regularization, the accuracy of linear models is shown to be improved significantly to become comparable to the more complex nonlinear algorithm. We find that none of the models or fingerprints were able to accurately predict the tensile modulus E, which we hypothesize is due to heterogeneity in data and data sources, as well as inherent challenges in measuring it. Finally, QSPR models revealed that the fraction of rotatable bonds, and the rotational degree of freedom affects polyamide properties most profoundly and can be used for back of the envelope calculations for a quick estimate of the polymer attributes (glass transition temperature, melting temperature, and density). These QSPR models, although having slightly lower prediction accuracy, show the most promise for the polymer chemist seeking to develop an intuition of ways to modify the chemistry to enhance specific attributes.

## 1. Introduction

Polyamides are a family of polymers that contain repeat units linked by amide groups. They are often prepared by hydrolytic polymerization, anionic polymerization, or solid phase synthesis. The materials exhibit desirable properties, such as high temperature resistance, high strength, good fatigue resistance, water absorption, chemical stability, and excellent wear behavior [1,2,3,4]. Silk and wool are two of the naturally occurring polyamides, while synthetic routes have resulted in materials that are ubiquitous in our daily use such as Kevlar, nylon, etc. The diversity and favorable properties of polyamides have resulted in them finding applications [4] in flexible packaging, automotive industries, and garments [5].

This diversity of attributes makes developing novel polyamides to achieve the desired material properties a challenging task. High structural diversity of these materials, ranging from linear or aliphatic to semi-aromatic or aromatic leads to large variation in the structural space. Additionally, polyamides exhibit strongly non-linear and anisotropic structure property relations, which makes a targeted design of the experiment to systematically study the correlations between structure and property difficult. One method that enables modeling of high dimensional and non-linear datasets and extracting information in machine learning is the focus of this study.

On one hand, having a diverse set of structures tests the limits of different types of fingerprints and the algorithm’s ability to capture patterns in this wide range. On the other hand, due to this wide range of chemical variations, there may be difficulties with finding these patterns, especially for those properties with fewer data points, which could lead to overfitting and poor predictive models. Additionally, subtle differences could cause some artifacts in the predictions driven largely by a few outlying examples. Hence, the goal of this paper is to establish robust models, including methods and hyperparameters that can be used to predict polyamide properties with reasonable fidelity.

Cheminformatics has been extensively applied in the small molecule space, especially for biological applications such as drug discovery [6,7]. As the bioinformatics space has grown, the use of open databases with chemical structures and corresponding properties is enabling development of robust predictive models, especially for small molecule discovery.

In addition, there have been seminal works in advancing chemical structure data processing and analytics in the general materials space. For example, density functional theory (DFT) can be used to generate a wide library of synthetic property data on which models can be trained to predict electronic properties for high throughput screening [8]. These datasets have been used to advance understanding in molecular representations and inverse design [9,10]. Another application is synthesis planning, where small molecule reactions are encoded and used to either predict the outcome of reactions or find reactants and conditions that would make the product of interest (also called retrosynthesis) [11,12,13].

Databases of experimentally measured polymer properties are relatively small compared to other small molecules or drug molecules [14], which makes learning polymeric properties more difficult. Nonetheless, there have been efforts toward developing machine learning models that enable rapid prediction of various electronic, mechanical, and thermal properties of polymers. For example, some early work using quantitative structure property relationship (QSPR) fingerprints that account for connection points between different monomers have been used to build predictive models [15]. In addition, previous studies have developed tools to allow for property prediction through online platforms [16,17]. To overcome the small dataset issue, transfer learning has recently been applied to predict thermal conductivity, where only a few experimental data were available, and this method helped to discover new polymers of high thermal conductivity through generative models [18,19]. Furthermore, there is work in the conjugated semiconducting polymer and fullerene space, using machine learning and deep learning to predict properties of bulk heterojunction solar cells [20]. This study showed applications of graph convolution neural networks, concatenation of graph convolution fingerprints, and comparison of this method to random forest with Morgan fingerprints.

Various combinations of molecular representations and regression models can be used depending on the target properties; however, the effect of choosing specific molecular representations and models is still being investigated [14]. These combinations of molecular representations typically result in a lack of interpretability in many of the features as more complex chemical information is encoded which focus more toward developing models with higher accuracy. In addition, the complexity of models and fingerprints, necessitate the use of computers and advanced algorithms to identify polymers with required properties and rendering them inaccessible to chemists. In this work, we focus on predicting density, tensile modulus, glass transition temperature, and melting temperature for polyamides with a goal of providing heuristic methods for property prediction for chemists. We focus on these properties, as they are key components of the mechanical and thermal reliability of polyamides and have data availability in open databases. We compare effects of fingerprint and model complexity on prediction fidelity. Following this, we identify dominating features of a polyamide that affects its properties by developing models with interpretable features.

## 2. Materials and Methods

In this section, we describe our approach to (i) the collection of polymer data, (ii) representation of polymer structures (SMILES, ECFP, CI, and QSPR), (iii) machine learning algorithms used to connect the input information through the polymer representations to predict the outputs (LR, SVM, RF), and finally (iv) metrics to assess the accuracy of these algorithms.

### 2.1. Data Collection

Polyamide structure and property data were manually collected from the PoLyInfo polymer database, an open data source maintained by the National Institute for Materials Science in Japan [21] in June 2020. The total data counts from this collection process are shown in the Gathered Points column of Table 1.

In this work, we focus on neat resins, and materials with multiple property measurements were represented by a single averaged value, leading to the total data counts shown in the Final Points column of Table 1. Details of this can be found in the Supplementary Information. This work focuses on four different properties: density in g/cc (ρ), tensile modulus in GPa (E), glass transition temperature in °C ($T_{g}$), and melting temperature in °C ($T_{m}$). The values of E and ρ in PoLyInfo are mostly measured at temperatures between 20−30°C, so the temperature dependence may not account for large discrepancies found in certain data.

### 2.2. Polymer Representation

Here, we describe one qualitative method for describing polymers and four quantitative descriptors of the polymer.

#### 2.2.1. Simplified Molecular-Input Line-Entry System (SMILES)

Polymer structure data were transcribed from images into a simplified molecular-input line-entry system (SMILES) strings for each structural repeating unit, each with 2 dummy atoms indicated the head and tail where the chain would continue (labeled as *) [22]. For use as inputs into predictive models, SMILES strings were translated into four distinct numerical representations.

#### 2.2.2. Extended Connectivity Fingerprint (ECFP)

**Figure 1:**
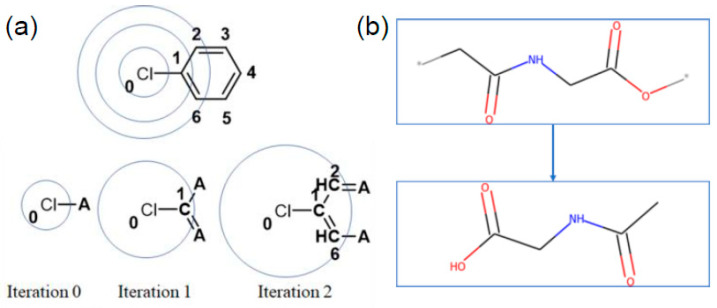
(**a**) Example of a chlorine atom in chlorobenzene finding its neighboring atoms to define chemical environment for an ECFP with radius 2. Reproduced from Lee [20]. (**b**) Example of hydrogen capping a monomer prior to calculating its ECFP.

The most commonly used numerical representation type of fingerprints used in the literature, and a focus of this paper, is the extended connectivity fingerprint (ECFP) [23]. ECFP, also known as the Morgan fingerprint, is a form of circular fingerprint that considers each atom’s properties and its neighboring environment up to r atoms away for a defined radius r. The radius here refers to the number of nearest neighbors, based on bonds, away from each atom, an illustration of this is provided in Figure 1a. For this study, we chose radius 2 and radius 10, later referred to as ECFP2 and ECFP10, respectively. Radius 2 was chosen because it is the convention used in most studies. Radius 10 was chosen because it was the value at which the fingerprint uniqueness was no longer increasing with increasing radius and will be addressed in Section 3.2. This uniqueness was calculated for each property as the number of unique fingerprints divided by the total number of data points (Final Points in Table 1). For each structure, the monomer is capped with a hydrogen atom at the * locations prior to calculation of the ECFP vector, shown in Figure 1b. The resulting ECFP is a 2048-bit vector; in this work, we calculate ECFPs using the RDKit software tool [24].

#### 2.2.3. Connectivity Indices

We use the connectivity indices (CI), as described by Bicerano in his seminal polymer quantitative structure–property relation work [15]. These indices are a basic embodiment of graph theory that describe the electronic environment and bonding configuration of the heavy (non-hydrogen) atoms in each repeating unit. Bicerano extended the connectivity indices for small molecules to describe polymers by providing information about atoms in neighboring units. The result is a set of four indices based on simple descriptors, such as atomic numbers, bond degrees, and numbers of valence electrons.

#### 2.2.4. QSPR Descriptors

The final numerical representation used in this work are these descriptors, which have the advantage of interpretability, and the ones we explored in this work are detailed in the Supplementary Information. Some examples include number of heavy atoms, number of hydrogen bonding groups, and the number of aromatic rings. These descriptors were calculated using RDKit, whether directly as a counter (e.g., for aromatic rings) or as an enabler for a self-coded counter (e.g., for Bicerano’s definition of rotational degrees of freedom, abbreviated as rotational DOFs). These descriptors account for the continuity of the polymer chain at the ends of the monomer structure. The resulting fingerprint is a one-hot encoded vector of these descriptors. These vectors can be further manipulated by encoding information about the CI and/or normalization of the descriptors. We normalize these descriptors by dividing the values by the number of heavy atoms; in the normalized feature set, the feature that is the number of heavy atoms is omitted, as all of them would have a value of 1. We expect these fingerprints to be less descriptive than ECFP; however, they are easily interpretable and can lead to back of the envelope calculations for properties, and hence, can help a polymer chemist develop an intuition for structural changes that lead to desired attributes.

### 2.3. Machine Learning Algorithms

In this section, we describe the algorithms used to establish the mapping between the input polymer representations described in Section 2.2 with the output polymer attributes of interest (density, glass transition temperature, melting temperature and modulus).

A few different machine learning algorithms, as implemented in the scikit-learn set of tools [25], are used to build models for polymer property prediction.

#### 2.3.1. Multiple Linear Regression (LR)

Multiple Linear Regression (LR) based on ordinary least squares, is used for the comparison as the baseline benchmark. This algorithm takes each feature independently and assigns coefficients. This work uses the Linear Regression function within scikit-learn to build the models. The default parameters are used.

#### 2.3.2. Support Vector Machine (SVM) Regression

Support Vector Machine (SVM) Regression is a method that uses decision boundaries to find an optimal hyperplane in the feature space [26]. This work uses the SVR function within scikit-learn to build the models. Most of the default parameters are used (e.g., radial basis function kernel), except for C = 20.0 and epsilon = 0.2. This C parameter is set to a higher value than the default (1.0) to increase the accuracy of the models, but not too high as to overfit more compared to that of the default value.

#### 2.3.3. Random Forest (RF)

Random Forest (RF), a method relying on an ensemble of predictions from various decision trees, is used [27]. This work uses the Random Forest Regressor function within scikit-learn to build the models. Most of the default parameters are used, except for the number of estimators (n_estimators), which is set to 10.

### 2.4. Machine Learning Accuracy Metrics

To assess the accuracy and generalizability of the models, we use k-fold cross-validation with k=5 [28]. This means that 80% of the input data is used for training and 20% is used for testing in 5 different runs, with each test set containing unique set of data points each time. Each model will yield an $R^{2}$ and an RMSE for its train and test sets. For method comparison, $R^{2}$ and RMSE values for the test sets were averaged over these 5 models. The KFold function in scikit-learn was used to create the folds, and the r2_score and mean_squared_error functions were used to calculate $R^{2}$ and RMSE values, respectively (RMSE is calculated as the positive square root of the result of mean_squared_error).

## 3. Results and Discussions

Our results in this section are organized as follows: (i) using the ECFP2 representation in conjunction with the SVM, LR, RF algorithms, (ii) comparing ECFP2 and ECFP10 for predicting properties (iii) shedding light on issues with predicting tensile modulus, (iv) enhancing interpretability of features using QSPR. This text will focus on presenting data for $T_{g}$ and the corresponding details for $T_{m}$, ρ, and E can be found in the Supplementary Information.

### 3.1. ECFP2 Representation Models

**Figure 2:**
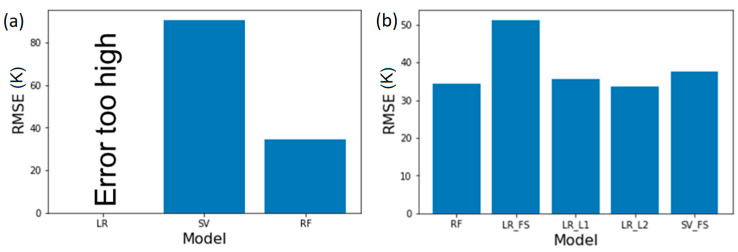
Test RMSE values for $T_{g}$ predictions using ECFP2 (**a**) comparison of LR, SV and RF; clearly, RF is significantly more accurate than LR and SV. One of the reasons is that for all properties, LR severely overfits and thus has too high of an error to display. (**b**) Post regularization, even linear models achieve accuracies that rival RF. FS, L1, and L2 here correspond to feature selection, L1 regularization, and L2 regularization, respectively.

**Figure 3:**
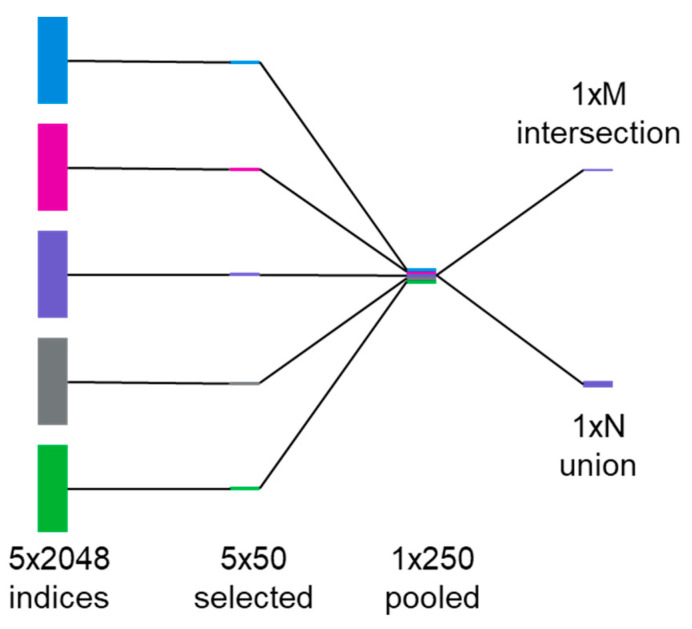
Process for random forest (RF) feature selection, starting by finding 50 out of 2048 most important indices of ECFP using 5 different RF models and returning the intersection and union sets of the pooled indices.

We implement ECFP2 in conjunction with LR, SVM, and RF to model polyamide properties. Through comparison of the RMSEs of model predictions of $T_{g}$, depicted in Figure 2a, we first observe that simple linear regression results in large RMSE, error that is several orders of magnitude larger than the property values (e.g., >10^10^ °C for $T_{g}$, which are typically <10^3^ °C); these are likely based on the limitation of the software. Upon using SVM, the RMSE is lower than for linear regression, but is still significant. Finally, leveraging RF, an intrinsically non-linear algorithm, provides the lowest RMSE and an accurate model. We hypothesize that this result is not due to the non-linear nature of the property, rather due to the large dimensionality of ECFP2, which leads to difficulties in using them as inputs for LR or SVM models. For ECFP2, the number of features (2048) is much greater than the number of data points for any of the properties. Meanwhile, RF models overcome this by selecting a subset of features while also providing estimates of variable importance based on the splitting done by its trees [27]. To use ECFP2 more effectively with LR and SVM, we first train a set of 5 RF models (80% train, 20% test) for a given property and record the top 50 most important features from each one; in this case, the features are indices within the ECFP2 bit vectors. We then take the intersections and unions of these top 50 lists to construct final index lists for use in feature selection prior to use as input into LR and SVM (Figure 3). The final number of features used in each case is listed in Table 2.

Alternatively, to enhance the accuracy of LR with ECFP, we use Least Absolute Shrinkage and Selection Operator (LASSO) LR [29] and Ridge regression [30], corresponding to LR with L1 and L2 regularization, respectively; the regularization hyperparameter was optimized for each property/fingerprint. Regularization here helps to decrease the coefficients associated with certain inputs to reduce the complexity of the model.

Figure 2b depicts the model RMSE for glass transition temperature post feature selection using above mentioned methods. We observe that by using RF based feature selection, the RMSE decreases for linear regression to ~50. For SVM, the RMSE decreases from 85 to 40 resulting in a significantly more robust model. By utilizing regularization methods, the RMSE is further reduced and both LASSO and Ridge regression with linear methods result in RMSE of ~35 which is similar to that of RF method. This suggests that a non-linear model such as RF is not necessary for an accurate $T_{g}$ prediction, however, the feature selection is critical to improve predictions when using fingerprints such as ECFP2 which results in bit vectors with large number of elements for each polymer.

### 3.2. Effect of Varying ECFP Radius

**Figure 4:**
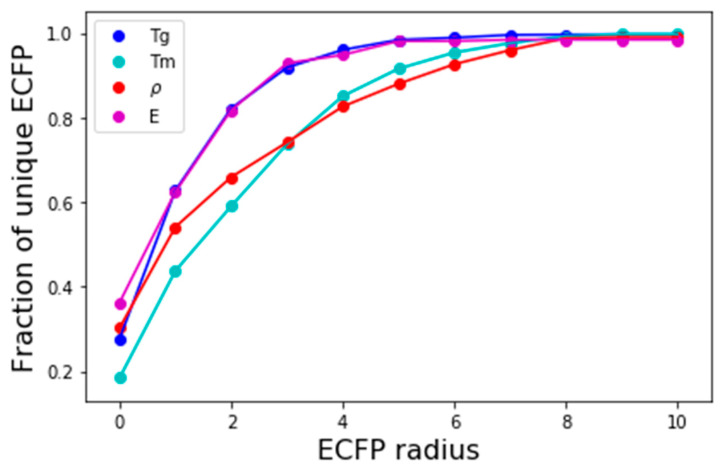
Comparison of the fraction of polyamides uniquely represented by varying the radius of ECFP in dataset for each of the property. Beyond ECFP 8, the value all polyamides can be distinctly represented.

**Figure 5:**
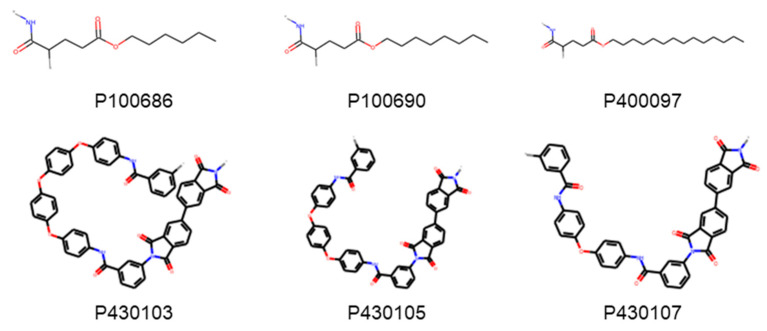
Two examples of polymers that cannot be distinguish by ECFP2 and need ECFP 8 to distinguish. The first example is of alkanes with increasing chain length, while the second is of difference in repeating ring patterns.

The number of polymers that can be mapped into unique bit-vectors depend on the ability of the fingerprinting technique to distinguish minor variations in structure and chemistry of the polymers. To explore this effect, we compare the effect of changing the radius of ECFP from 0 to 10 and comparing model accuracies. Figure 4 depicts the fraction of polyamides in the density dataset that are uniquely represented by ECFP for the corresponding radius. For a radius of 0; only 30% are represented, this rises quickly and at a radius of 2, 70% of the polyamide’s chemistries are captured. Upon extending the radius to 8, close to 100% of the polyamides are unique expressed using ECFP. At low values of ECFP radii, the two most common polymers that are hard to distinguish are (1) those differing by length of an alkyl chain, and (2) those differing by repeating ring patterns. Figure 5 depicts examples of two of the polyamides that cannot be distinguished by small radius of ECFP and need radii of 8 to discern as the difference in structure is minor.

To compare effect of ECFP radius, we train the RF model and compare the test R^2^ values. The details of this are shown in Table S4. For smaller radii, we average our properties of polyamides that are not differentiable by ECFP of that corresponding radius. Table S4 in Supplementary includes data to compare prediction accuracies for ECFP radii 2 and 10 and we find that the two cases do not show much difference; hence, we conclude that ECFP2 is capable of accurately predicting $T_{g}$, $T_{m}$, and ρ when combined with feature selection and focus on ECFP2 for the remainder of the discussion. Next, we turn our attention to tensile modulus predictions.

### 3.3. Inability to Accurately Predict Tensile Modulus

When training models to predict E for polyamides, we find that none of the models and none of the descriptors lead to accurate predictions with RMSE being larger than 1 GPa. This is puzzling, as ECFP can predict static properties fairly accurately such as density and material properties such as $T_{g}$ and $T_{m}$. Varying fingerprints, machine learning models, and feature selection did not result in more accurate models. This could be due to critical information regarding the gradient of internal energy not being captured by either fingerprints or models. Alternately, we hypothesize that this critical result could be due to variation in testing methods, anomalous properties of some polyamides such as Kevlar, or microphase separation which can result in heterogeneity resulting in data not being fit for machine learning. One such example could be variability in temperature and strain rates when performing the test which has been demonstrated to have significant effect on fiber filled polyamide modulus [31]. In addition, the hierarchical structures of the polymer samples may impact their ultimate measured properties [32,33], which can lead to difficulties when predicting such properties. This highlights the need for consistent standards for data storage, exhaustive metadata, novel fingerprints, or machine learning methods that could capture such properties.

In the remainder of the paper, we will focus on $T_{g}$, $T_{m}$, and ρ for polyamides and identify molecular features of polyamide that would enable back of the envelope calculations for properties.

### 3.4. QSPR Models for Interpretability

A primary goal of this work is to develop interpretable models that can serve as a guide to polymer chemists. ECFP due to its complex nature, is challenging to interpret and therefore cannot provide guides to visual distinction of polymers. To develop such models, we leverage physically interpretable QSPR descriptors as mentioned in the methods section. Furthermore, we can add connectivity indices (CI) and/or normalize the QSPR descriptors to increase accuracy while maintaining interpretability.

First, comparing models for $T_{g}$, RF with QSPR fingerprints gives an RMSE of 39.6 when compared to 33 for ECFP. The marginally higher RMSE suggests that this simplistic QSPR representation, which lacks the structural detail of ECFP, can predict glass transition temperatures with decent accuracy. Upon addition of CI and normalization, the RMSE decreases to 39.3 giving marginally better models. When using SVM; the RMSE is found to be 44 and is 33% higher than ECFP with RF. This does not improve further upon addition of CI and normalization. Finally, linear regression models, which are most attractive for interpretability, are less accurate with RMSE of ~50 when using QSPR, QSPR with CI, or normalized QSPR + CI. This suggests that a simple QSPR feature set with a non-linear RF model can be used to predict glass transition temperature for polyamides with relatively high accuracy. Upon using SVM and linear regression, the RMSE is higher; however, they still result in usable models.

**Figure 6:**
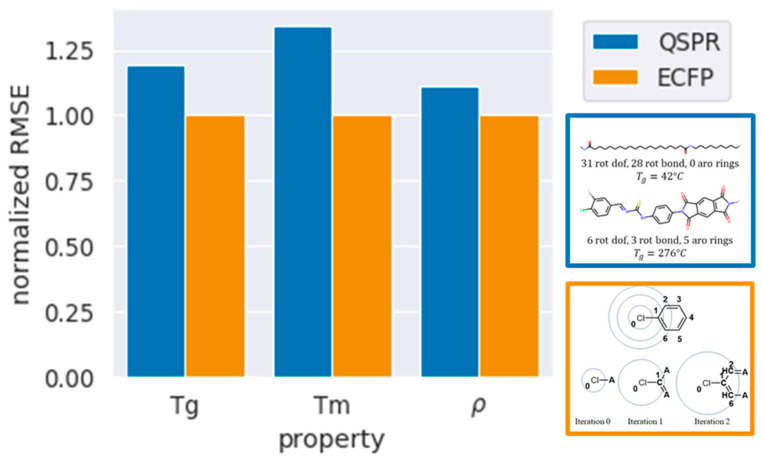
Summary of the best models using each fingerprinting technique represented as normalized RMSE. The normalization is performed relative to the most accurate ECFP model.

We find similar trends for predictions of $T_{m}$ and ρ. QSPR models result in higher RMSE when compared to ECFP, with $T_{m}$ having 27% higher RMSE, and ρ being fairly accurate with only a 10% higher RMSE. RF gives the lowest RMSE, followed by SVM, and finally LR. Upon addition of CI fingerprints and normalization, the RMSE decreases by a minimal amount. A comparison of the RMSE between QSPR and ECFP is depicted in Figure 6, with the RMSEs normalized to those of ECFP models with lowest error. Table 3 further details the RMSE and R^2^ of the various models developed using QSPR, QSPR with CI, and normalized QSPR with CI. The overall comparison of all combinations of fingerprints and algorithms for each property can be found in the Supplementary Table S4.

### 3.5. Feature Importance and Examples

**Figure 7:**
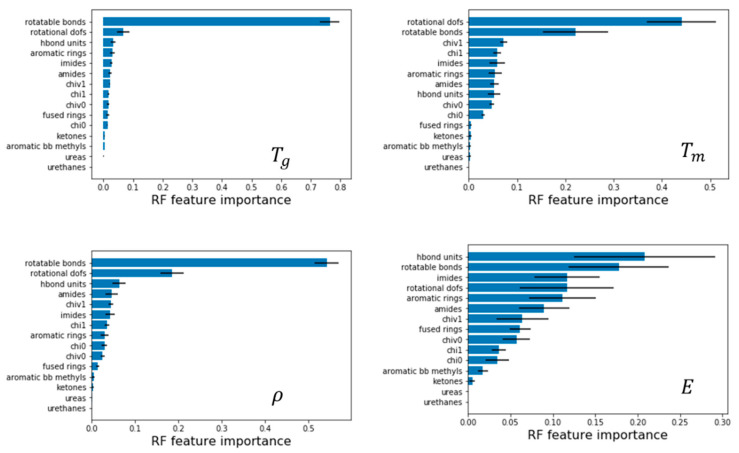
Feature importance of QSPR descriptors for models of $T_{g}$, $T_{m}$, ρ, and E calculated from RF models using normalized QSPR descriptors with CI as input.

We next leverage the QSPR models to identify what features are most critical to impart properties to polyamides by analyzing Gini feature importance using random forest. Here, we discuss examples for models with normalized QSPR features, as it is more evident which features are most important compared to models with unnormalized features. Figure 7 depicts these features for all properties; the bars correspond to mean values and the error bars correspond to the standard deviation, both results of aggregating multiple models from 5-fold cross validation. In the following examples, the unnormalized values are presented as well for clarity and ease of replication.

**Figure 8:**
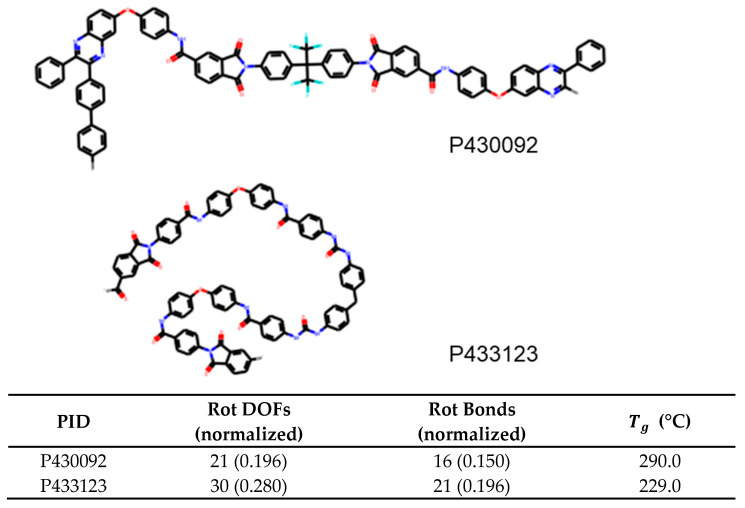
Example of two polyamides, with IDs P430092 and P433123, for which the number of rotatable bonds can be used to predict the trend of $T_{g}$ accurately.

For glass transition temperature, we find that the number of rotatable bonds has a feature importance of 0.764 and is at least 12 times more important than the next feature, which is rotational DOFs at 0.06. This is an important result and suggests that polyamides with a larger number of rotatable bonds will have lower $T_{g}$, and for quick prediction, the number of rotatable bonds can be used to differentiate polyamides. An example of this is shown in Figure 8. Here, the first molecule P430092 contains 0.150 rotatable bonds per heavy atom while P433123 contains 0.196. Due to the correlation demonstrated in this paper, a reasonable guess would be that $T_{g}$ of the first molecule is higher than the second. This in indeed the case with the glass transition temperature being 290°C and 229°C, respectively.

**Figure 9:**
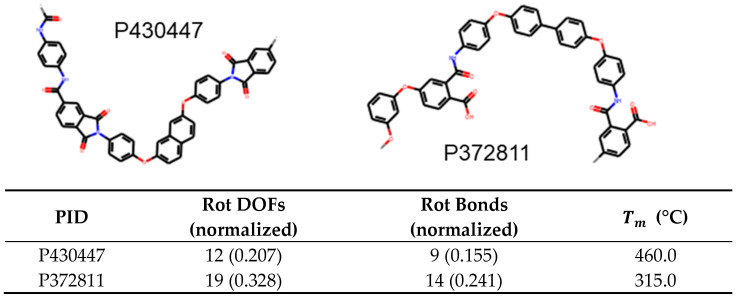
Example of two polyamides, with IDs P430447 and P3272811, whose melting temperatures can be predicted using trend in rotational DOFs.

For melting temperature, we find that rotational DOFs of a molecule has a feature importance of 0.44, a correlation weaker than what was found for $T_{g}$, nonetheless fairly significant. The melting temperature is found to decrease with increasing rotational DOFs. Next, rotatable bonds also show a negative correlation with a feature importance of 0.22 with other features being less than 0.1. An example of using this is shown in Figure 9. In this case, P430447 has 0.207 rotational DOFs per heavy atom while P372811 has 0.328; hence, we expect the former to have higher melting temperature. This is indeed the case as the former has a $T_{m}$ of 460°C while the latter has a $T_{m}$ of 315°C.

**Figure 10:**
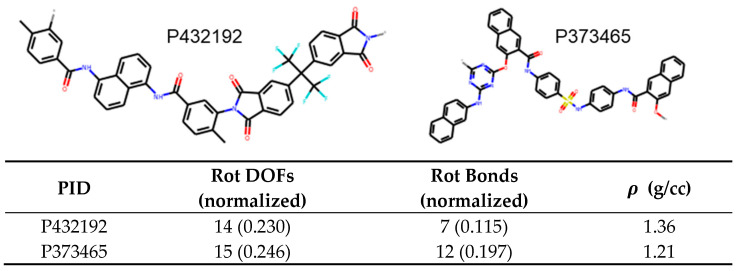
Example of two polyamides, with IDs P432192 and P373465, whose densities can be predicted using trend in rotatable bonds.

Finally, for density, rotatable bonds have the highest feature importance of 0.54 and decreases density, followed by rotational DOFs with 0.18 and increases density, all other feature importance was less than 0.1. An example of leveraging this is depicted in Figure 10. In this case, P432192 has 0.115 rotatable bonds per heavy atom while P373465 has 0.197. Therefore, we can predict that the latter molecule has lower density owing to the higher proportion of rotatable bonds. This is indeed the case with the densities being 1.36 g/cc and 1.21 g/cc, respectively.

**Figure 11:**
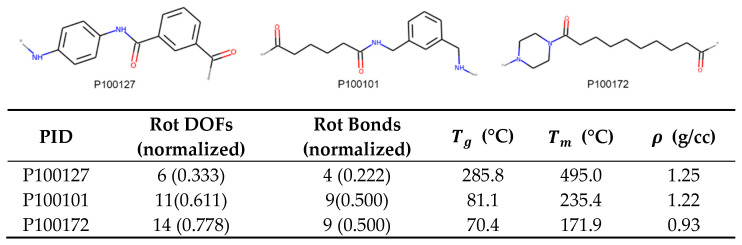
Example of three polyamides, with IDs P100127, P100101, and P100172, whose $T_{g}$, $T_{m}$, and ρ can be predicted using trends described in this section.

Figure 11 combines the results from this work to predict the trends of $T_{g}$, $T_{m}$, and ρ for P100101, P100127 and P100172. The table provides the rotational DOFs and rotatable bonds, which are the important features for these properties. We expect that due to higher number of rotatable bonds, P100172 and P100101 should have lower $T_{g}$ and $T_{m}$ than P100127. As P100172 and P100101 have the same number of rotatable bonds, P100172, with more rotational DOFs, should have lower $T_{g}$ and $T_{m}$ than P100101. Additionally, due to the trend of rotational DOFs, the density of P100172 should be lowest, followed by P100101, and finally, P100127 should be the highest. This is indeed true, as shown in the table with Figure 11. Therefore, we can rank order three polyamides using the two most important descriptors.

Finally, studying the three properties reveals that rotatable bonds and rotational DOFs are found as most critical parameters for predicting $T_{g}$, $T_{m}$, and ρ for polyamides. The examples provided help us perform quick screening to rank order these chemistries to perform rational design. For example, tuning the ratio of different components used when synthesizing polyamides to match these structural features, such as the number of rotatable bonds or the number of hydrogen bonding units, can lead to more advantageous properties for specific applications [34,35,36].

## 4. Conclusions

In this paper, we have developed machine learning models to predict $T_{g}$, $T_{m}$, and ρ for polyamides. We compare ECFP fingerprints with QSPR, and linear models with non-linear ones. We find that RF, a non-linear model, results in the most reliable prediction for $T_{g}$, $T_{m}$, and ρ when feature selection is not accounted for. When using ECFP, LR and SVM, we obtain high errors and models not useful for property predictions. However, when either feature selection using RF or regularization (L1 or L2) are implemented, the accuracy for ECFP LR models increases to become comparable to that of the RF model. This suggests that identifying the right subset of fingerprint feature set or appropriate weighting of different features even when using full feature set can lead to high fidelity prediction without the need for complex non-linear models.

To study this further, we develop QSPR type models and find that while accuracy decreases; these models can be used to predict properties of polyamide with high fidelity. Furthermore, this leads to identification of interpretable features that affect polyamide properties and develop heuristic comparison to enable rational design for chemists. Finally, we find that number of rotatable bonds and rotational DOFs are two properties that have the largest effect of polyamide properties. Furthermore, as this field grows and newer QSPR features are developed, we expect the model accuracies to increase further, and result in highly interpretable models that easy to implement and rival accuracies of more complex algorithms.

However, none of the models were effective in predicting the tensile modulus of polyamides; this opens questions on potential information that is not captured by simple fingerprints that is required to predict modulus. Potential methods to bridge this gap are multitask learning and transfer learning. Multitask learning aims to predict multiple properties simultaneously [37]; for properties with related features, the synergy helps boost the predictive accuracy of each property relative to many separate single task models. Meanwhile, transfer learning leverages information from a model to predict a certain property to help predict other properties, such as those that are more complex and/or have less data [38,39]. These methods will be the subject of future studies.

## Figures and Tables

**Table 1:** Data counts for each property, describing the number initially gathered from PoLyInfo and the number remaining after processing.

Property	Abbreviation	Gathered Points	Final Points
Density (g/cc)	ρ	1248	390
Tensile modulus (GPa)	E	809	306
Glass transition temperature (°C)	$T_{g}$	2072	1388
Melting temperature (°C)	$T_{m}$	1723	942

**Table 2:** Number of features in the intersection (Int) and union (Uni) sets for each property after the RF feature selection procedure.

Num of Features		Int	Uni
ECFP2	ρ	11	113
	E	17	112
	$T_{g}$	11	108
	$T_{m}$	16	99
ECFP10	ρ	6	135
	E	5	160
	$T_{g}$	13	123
	$T_{m}$	10	132

**Table 3:** The model fit data and the most important features for each of the properties using interpretable QSPR fingerprints. The most important features are based on normalized feature sets.

Method	Metric (Test)	ρ	E	$T_{g}$ (°C)	$T_{m}$ (°C)
LR	$R^{2}$	0.720	0.231	0.755	0.411
	RMSE	0.073	1.12	48.62	66.78
SVM	$R^{2}$	0.479	0.216	0.799	0.448
	RMSE	0.100	1.10	43.92	64.4
RF	$R^{2}$	0.721	0.194	0.833	0.421
	RMSE	0.073	1.15	40.03	65.94
	most important features	rot bonds rot dofs	hbond units rot bonds	rot bonds rot dofs	rot dofs rot bonds

## Data Availability

The data presented in this study are available on request from the corresponding author.

## Supplementary Materials

The following are available online at https://www.mdpi.com/article/10.3390/polym13213653/s1, Figure S1: title, Table S1: title, Video S1: title. Figure S1: Example of translating an image of a structural repeating unit into its corresponding SMILES string; Figure S2: Data distribution of thermal and mechanical properties of polyamides in PoLyInfo; Table S1: 5-fold test metrics before and after averaging values for RF models; Figure S3: Example of density value distributions for a set of unique ECFP2 that have multiple values; Figure S4. 5-fold linear regression result comparison between intersection (left) and union (right) sets using ECFP2 fingerprints for (a) density. (b) tensile modulus. (c) glass transition temperature. (d) melting temperature; Figure S5. 5-fold linear regression result comparison between intersection (left) and union (right) sets using ECFP10 fingerprints for (a) density. (b) tensile modulus. (c) glass transition temperature. (d) melting temperature; Figure S6. 5-fold SVM result comparison between intersection (left) and union (right) sets using ECFP2 fingerprints for (a) density. (b) tensile modulus. (c) glass transition temperature. (d) melting temperature; Figure S7. 5-fold SVM result comparison between intersection (left) and union (right) sets using ECFP10 fingerprints for (a) density. (b) tensile modulus. (c) glass transition temperature. (d) melting temperature; Table S2. 5-fold validation test set metrics for linear regression models before and after using feature selection (FS) based on the intersection (Int) and union (Uni) feature sets; Table S3. 5-fold validation test set metrics for SVM models before and after using feature selection (FS) based on the intersection (Int) and union (Uni) feature sets; Figure S8. Comparison of 5-fold test RMSE for linear regression (LR) and SVM (SV) models with no feature selection versus models with feature selection using the intersection (I) and union (U) sets for different fingerprint/property combinations. (a) ECFP2/*ρ*. (b) ECFP10/*ρ*. (c) ECFP2/E. (d) ECFP10/E. (e) ECFP2/$T_{g}$. (f) ECFP10/$T_{g}$. (g) ECFP2/$T_{m}$. (h) ECFP10/$T_{m}$; Figure S9. Comparison of 5-fold test RMSE for random forest (RF) models versus for linear regression (LR) and SVM (SV) models with feature selection using the intersection (I) and union (U) sets for different fingerprint/property combinations. (a) ECFP2/*ρ*. (b) ECFP10/*ρ*. (c) ECFP2/E. (d) ECFP10/E. (e) ECFP2/$T_{g}$. (f) ECFP10/$T_{g}$. (g) ECFP2/$T_{m}$. (h) ECFP10/$T_{m}$; Table S4. Summary of the best R^2 and RMSE metrics for all representation/model/property combinations studied in this work; Spreadsheet S1: Polymer IDs and SMILES strings for all polyamides used in this study.
