# Supplementary material for: Comparison of Machine Learning Methods towards Developing Interpretable Polyamide Property Prediction
Source: Polymers (Basel). 2021 Oct 23;13(21):3653. doi: 10.3390/polym13213653 (PMC8587315; doi:10.3390/polym13213653)
Supplement: Supplementary file 1 [file polymers-13-03653-s001.zip › polymers-1434412-supplementary.pdf]

Details of data gathering and storage

The workflow to capture and stope polymers into a machine readable format is described below:

1. Identifying the polymer identification (PID) field in PolyInfo and using that as a label.
2. Translating the structural repeating unit image into a string following the simplified molecular-input line-entry system (SMILES) convention [1], using * to indicate connection points, as shown in Figure S1, for use in subsequent molecular operations, such as fingerprinting. The full list of PIDs and SMILES strings is attached as a separate supplementary CSV file.
3. Finding data tables for each property of interest and recording for each data point:
    a. Sample ID
    b. Material Type
    c. Additives
    d. Property [units]
    e. Method
    f. Condition

4. Processing polymer properties prior to use in predictive models. The raw data from PoLyInfo yields distributions of measured property data for each polymer. First, we filter the data by selecting only samples labeled as "Neat resin" in the Material Type column. For polymers that only had one measured value left, that was used as the representative value. For those that had a distribution of measurements left, these distributions were consolidated into single representative values; here, the mean values of the distributions are used. We

excluded data which has standard deviation in reported values larger than 30°C for T_g and T_m. The final dataset sizes after consolidation are shown in Figure S2.

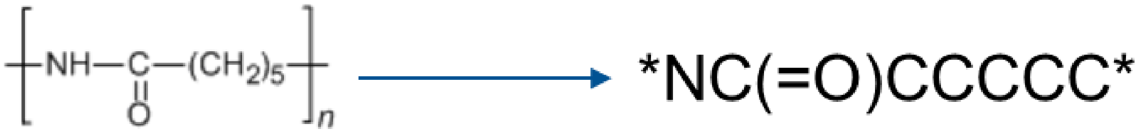

Figure S1. Example of translating an image of a structural repeating unit into its corresponding SMILES string.

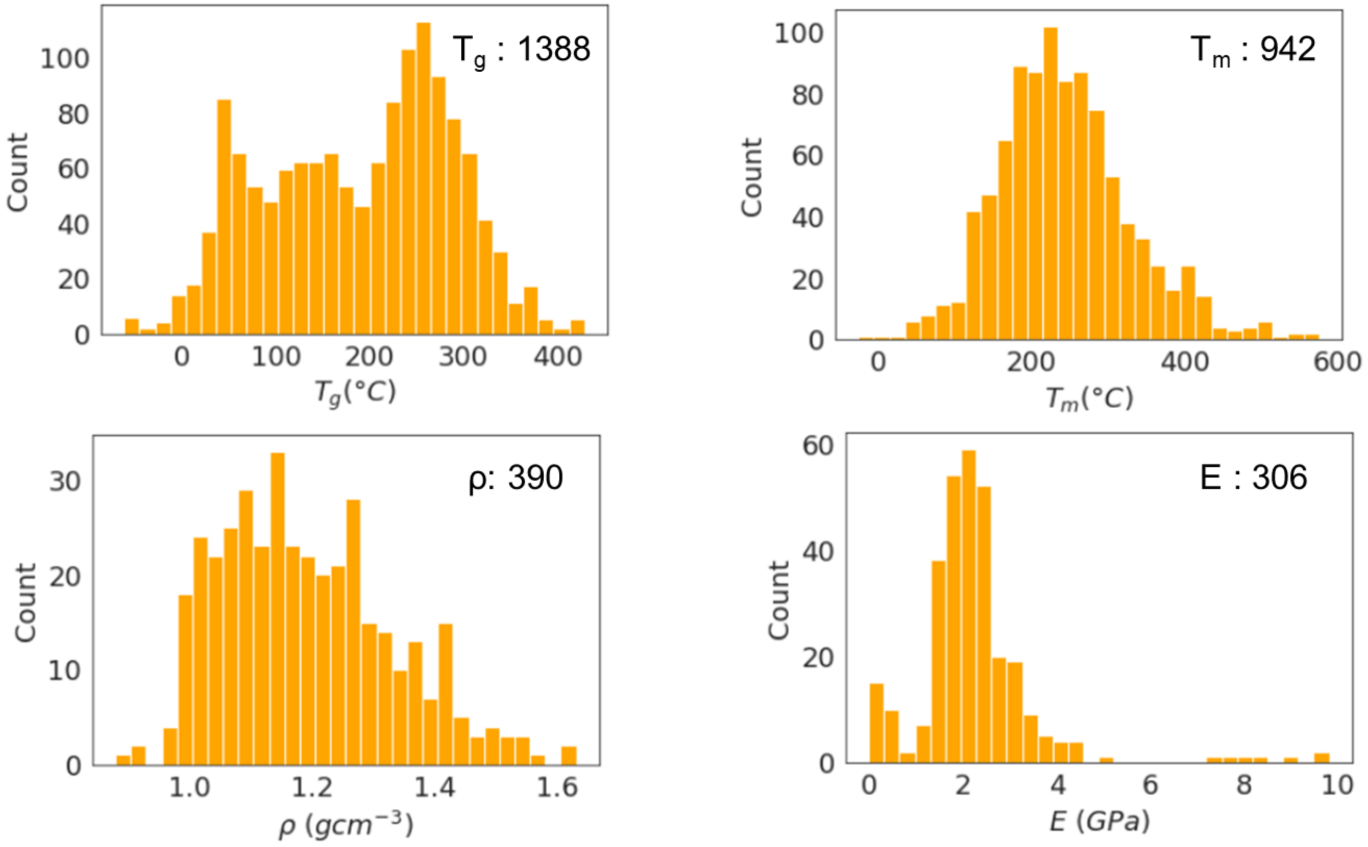

Figure S2. Data distribution of thermal and mechanical properties of polyamides in PoLyInfo.

Effect of fingerprint uniqueness

A comparison of metrics for models using ECFPs as inputs is shown in Table S1. Here, average refers to taking the average property value for each unique ECFP rather than the conventional way, where there could be different values corresponding to the same ECFP. Due to the variability in the distribution of a property for a given ECFP (e.g. in Figure S3), it is expected that averaging would cause some error.

Table S1. 5-fold test metrics before and after averaging values for RF models.

	**ECFP2**		**ECFP2 avg**		**ECFP10**		**ECFP10 avg**	
**Property**	**R2**	**RMSE**	**R2**	**RMSE**	**R2**	**RMSE**	**R2**	**RMSE**
ρ	**0.69**	**0.08**	0.56	0.08	0.67	0.08	**0.69**	**0.08**
E	**0.30**	**1.03**	0.18	1.21	0.15	1.17	**0.20**	**1.16**
T_g	**0.88**	**34.4**	0.83	38.7	**0.84**	**39.5**	0.83	40.2
T_m	**0.66**	**50.6**	0.55	63.5	0.63	52.5	**0.66**	**50.8**

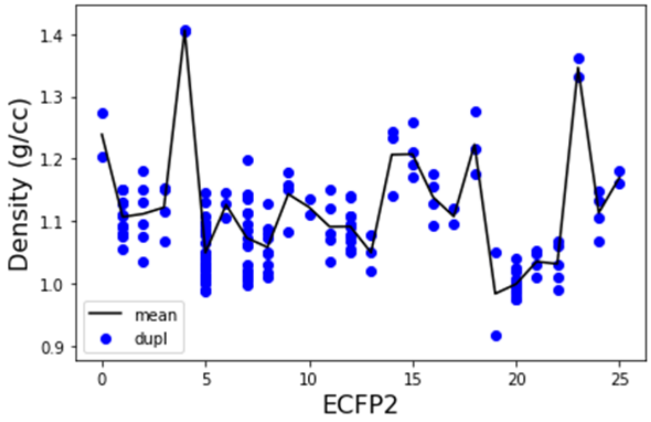

Figure S3. Example of density value distributions for a set of unique ECFP2 that have multiple values.

Effect of feature selection

To figure out which indices of the ECFP2 and ECFP10 fingerprints were most important for inference, five RF models were trained for each property and fingerprint combination (e.g., density and ECFP2) and the feature_importances_ attribute from scikit-learn was extracted [2]. For each model, the 50 most important indices were compiled into a list. Once these indices were identified, the intersection and union sets were determined from the five lists for each combination. Figures S4-S7 show the effect of choosing the intersection versus choosing the union for different algorithm/fingerprint combinations. The final test metrics are listed in Table S2 and Table S3 for LR and SVM respectively to compare model accuracy before and after feature selection. Figure S8 shows the RMSE comparisons in Table S2 and Table S3 graphically. Figure S9 shows the RMSE comparisons between models using feature selection versus just using RF directly.

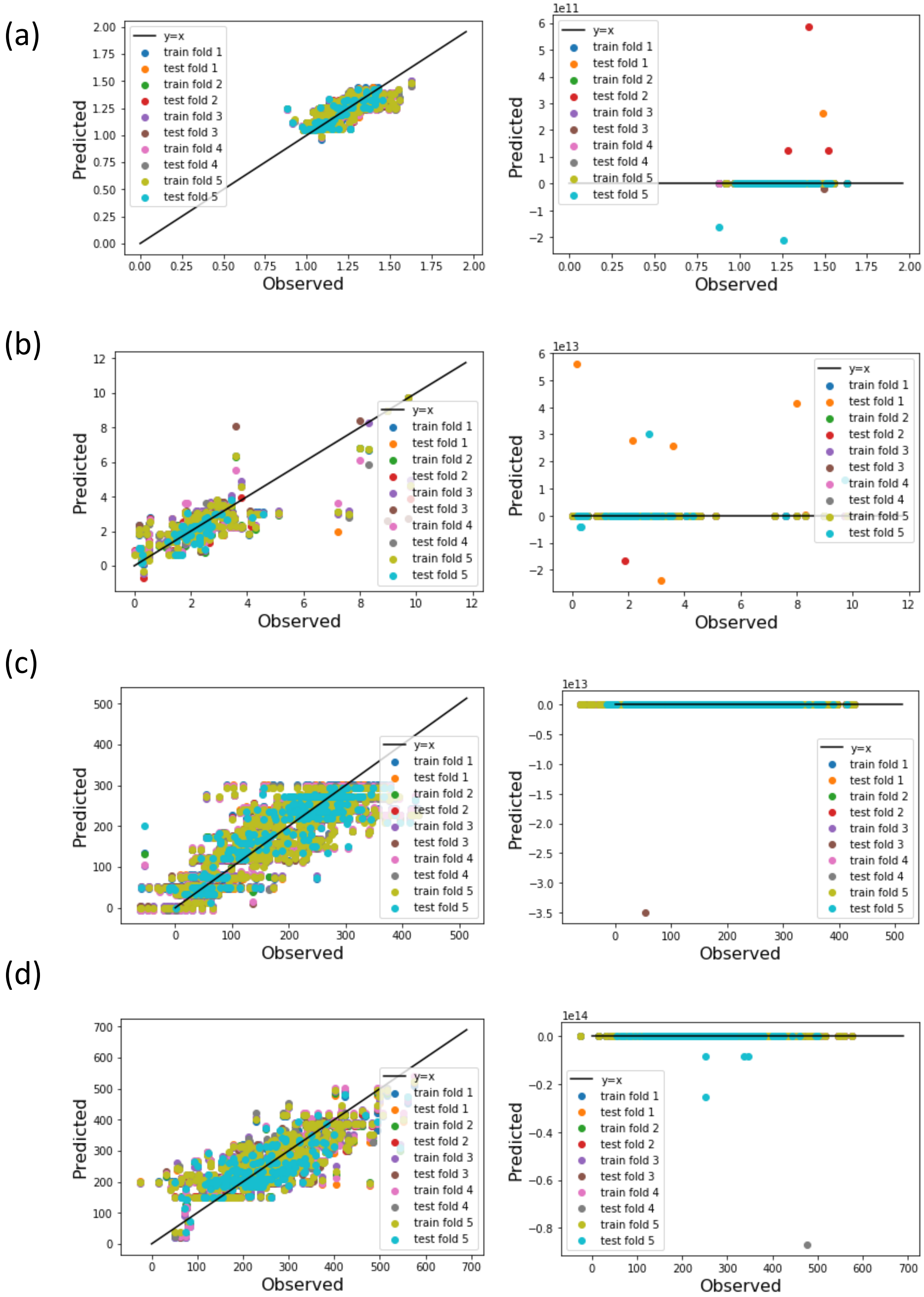
(a)
(b)
(c)
(d)
Predicted
Observed
y=x
train fold 1
test fold 1
train fold 2
test fold 2
train fold 3
test fold 3
train fold 4
test fold 4
train fold 5
test fold 5
1e11
1e13
1e13
1e14

Figure S4. 5-fold linear regression result comparison between intersection (left) and union (right) sets using ECFP2 fingerprints for (a) density. (b) tensile modulus. (c) glass transition temperature. (d) melting temperature.

(a)

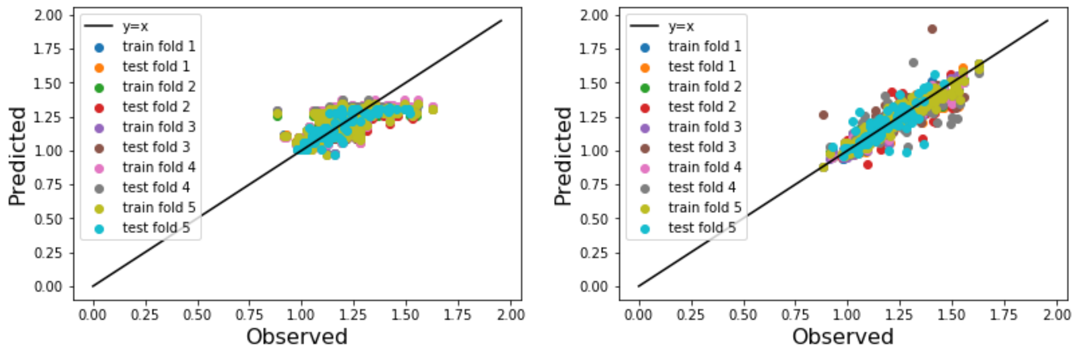

y=x
train fold 1
test fold 1
train fold 2
test fold 2
train fold 3
test fold 3
train fold 4
test fold 4
train fold 5
test fold 5
Predicted
Observed

(b)

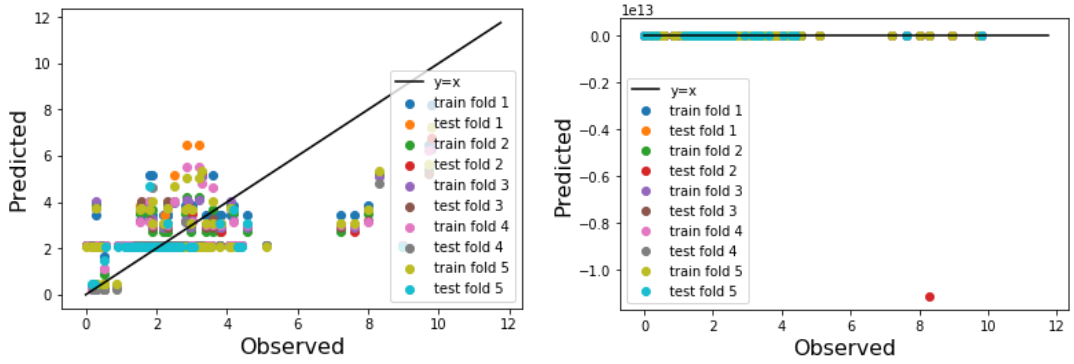

1e13
y=x
train fold 1
test fold 1
train fold 2
test fold 2
train fold 3
test fold 3
train fold 4
test fold 4
train fold 5
test fold 5
Predicted
Observed

(c)

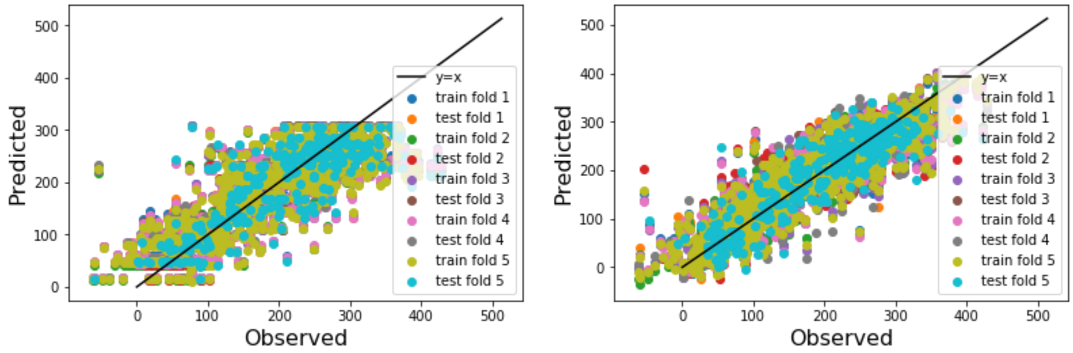

y=x
train fold 1
test fold 1
train fold 2
test fold 2
train fold 3
test fold 3
train fold 4
test fold 4
train fold 5
test fold 5
Predicted
Observed

(d)

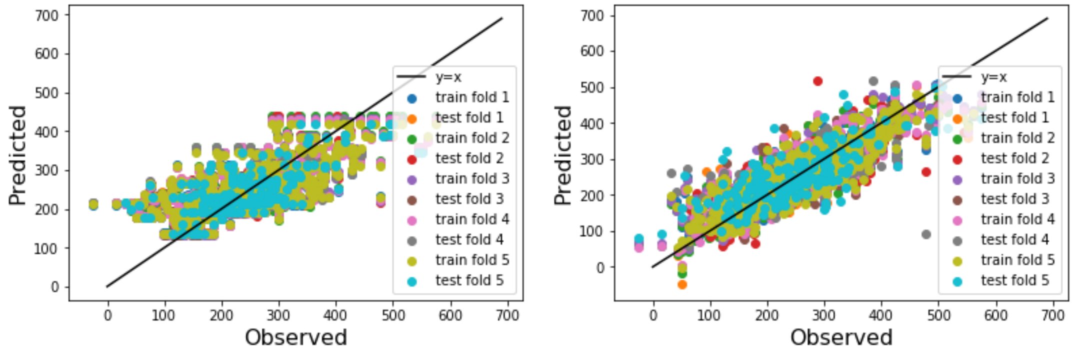

y=x
train fold 1
test fold 1
train fold 2
test fold 2
train fold 3
test fold 3
train fold 4
test fold 4
train fold 5
test fold 5
Predicted
Observed

Figure S5. 5-fold linear regression result comparison between intersection (left) and union (right) sets using ECFP10 fingerprints for (a) density. (b) tensile modulus. (c) glass transition temperature. (d) melting temperature.

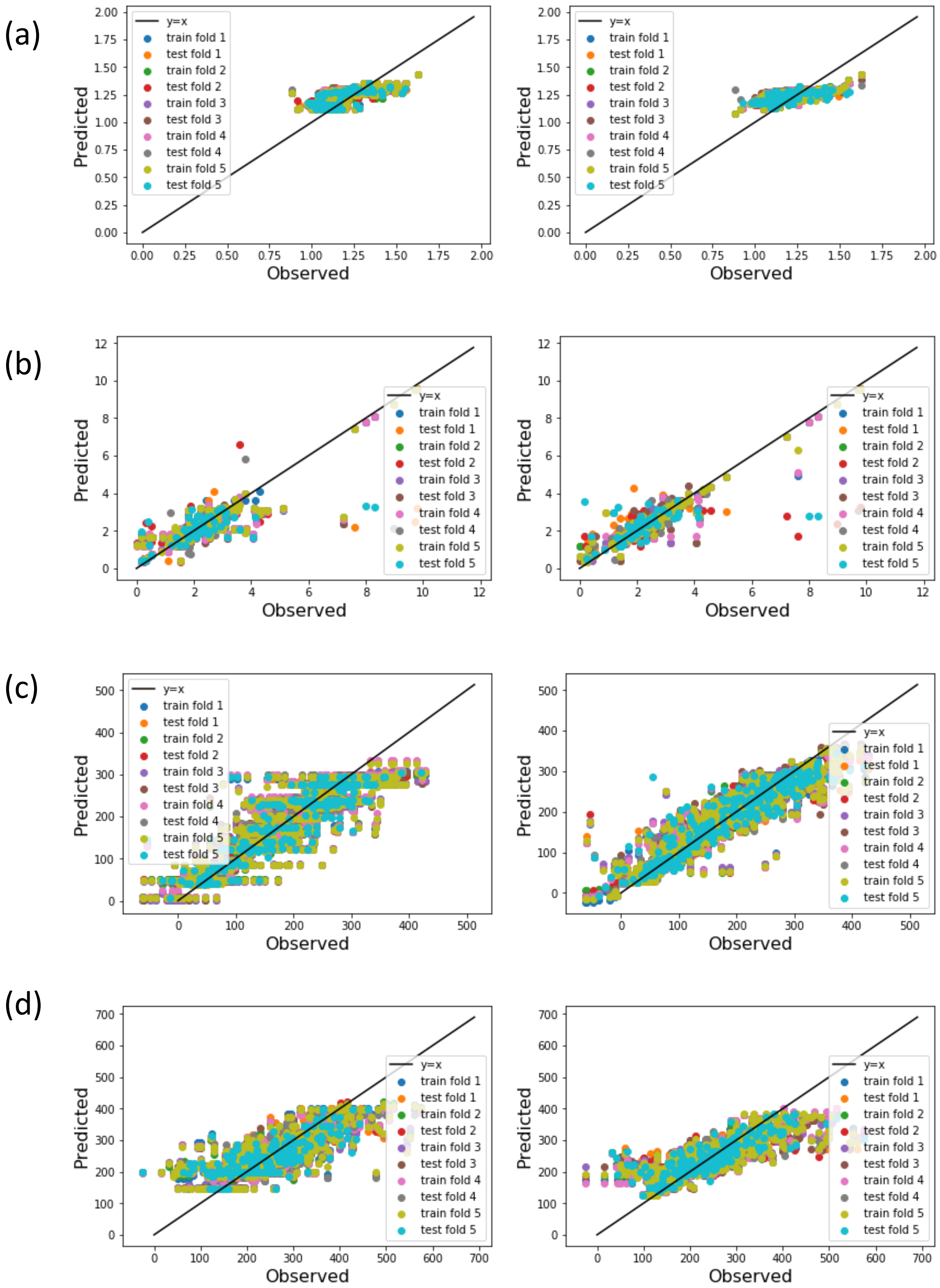
(a)
(b)
(c)
(d)
y=x
train fold 1
test fold 1
train fold 2
test fold 2
train fold 3
test fold 3
train fold 4
test fold 4
train fold 5
test fold 5
Predicted
Observed

Figure S6. 5-fold SVM result comparison between intersection (left) and union (right) sets using ECFP2 fingerprints for (a) density. (b) tensile modulus. (c) glass transition temperature. (d) melting temperature.

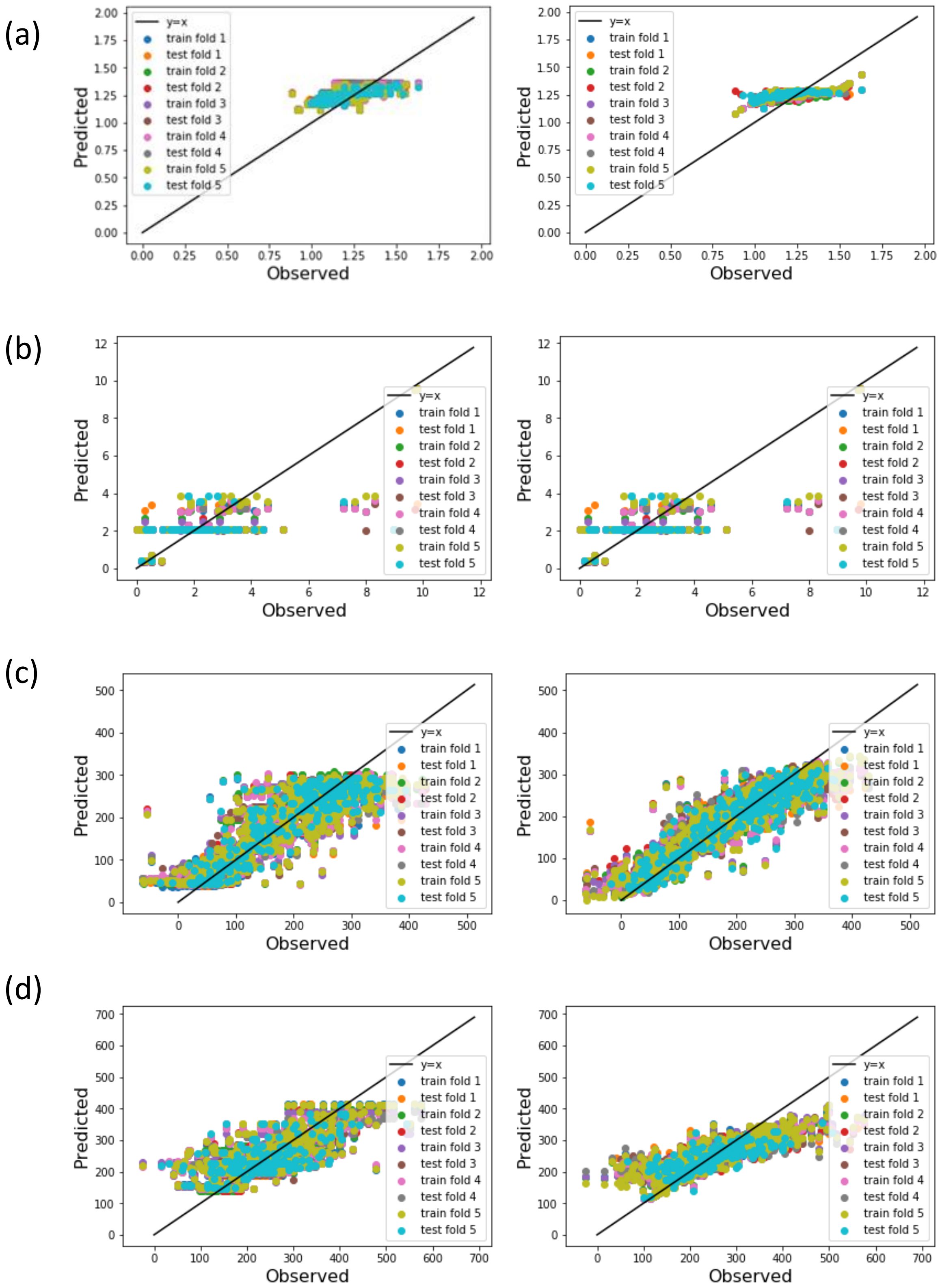

(a)
(b)
(c)
(d)
Predicted
Observed
y=x
train fold 1
test fold 1
train fold 2
test fold 2
train fold 3
test fold 3
train fold 4
test fold 4
train fold 5
test fold 5

Figure S7. 5-fold SVM result comparison between intersection (left) and union (right) sets using ECFP10 fingerprints for (a) density. (b) tensile modulus. (c) glass transition temperature. (d) melting temperature.

Table S2. 5-fold validation test set metrics for linear regression models before and after using feature selection (FS) based on the intersection (Int) and union (Uni) feature sets.

	**ECFP2 no FS**		**ECFP2 FS Int**		**ECFP2 FS Uni**		**ECFP10 no FS**		**ECFP10 FS Int**		**ECFP10 FS Uni**	
**Property**	**R2**	**RMSE**	**R2**	**RMSE**	**R2**	**RMSE**	**R2**	**RMSE**	**R2**	**RMSE**	**R2**	**RMSE**
ρ	n/a	n/a	**0.63**	**0.08**	n/a	n/a	n/a	n/a	0.50	0.09	0.58	0.09
E	n/a	n/a	**0.33**	**1.02**	n/a	n/a	n/a	n/a	0.09	1.12	n/a	n/a
T_g	n/a	n/a	0.73	51.2	n/a	n/a	n/a	n/a	0.70	53.4	**0.79**	**44.7**
T_m	n/a	n/a	0.56	57.8	n/a	n/a	n/a	n/a	0.45	64.5	**0.57**	**56.8**

Table S3. 5-fold validation test set metrics for SVM models before and after using feature selection (FS) based on the intersection (Int) and union (Uni) feature sets.

	**ECFP2 no FS**		**ECFP2 FS Int**		**ECFP2 FS Uni**		**ECFP10 no FS**		**ECFP10 FS Int**		**ECFP10 FS Uni**	
**Property**	**R2**	**RMSE**	**R2**	**RMSE**	**R2**	**RMSE**	**R2**	**RMSE**	**R2**	**RMSE**	**R2**	**RMSE**
ρ	0.21	0.12	**0.35**	**0.11**	0.27	0.11	n/a	0.14	0.23	0.12	0.10	0.13
E	0.35	1.01	0.28	1.06	**0.38**	**0.99**	0.27	1.08	0.15	1.16	0.28	1.06
T_g	0.77	47.6	0.79	45.4	**0.85**	**37.6**	0.73	50.8	0.75	59.1	0.81	42.4
T_m	0.44	65.4	0.54	59.1	**0.55**	**58.0**	0.30	73.1	0.47	62.6	0.50	61.4

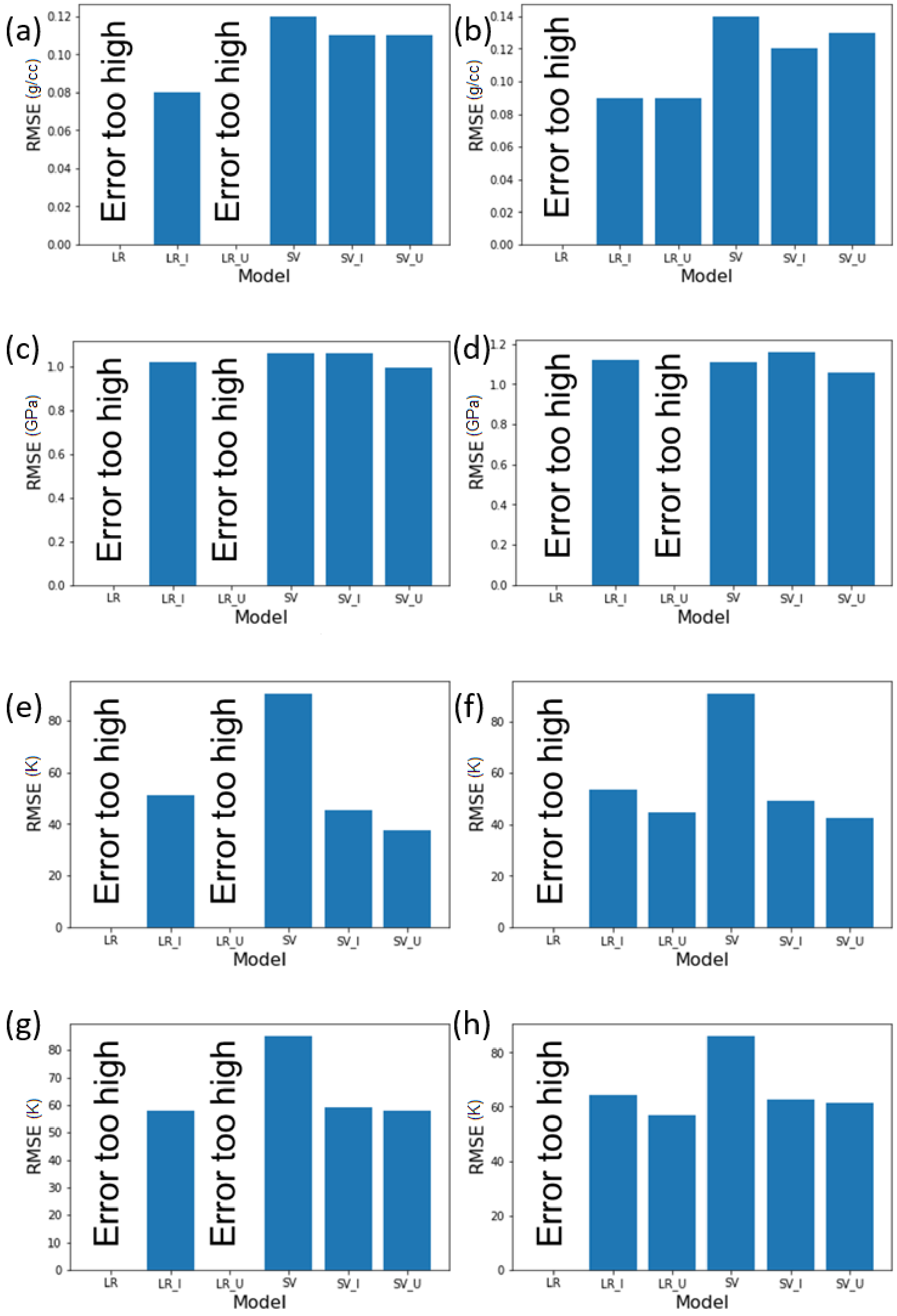
(a)
(b)
(c)
(d)
(e)
(f)
(g)
(h)
RMSE (g/cc)
RMSE (GPa)
RMSE (K)
Error too high
LR
LR_I
LR_U
SV
SV_I
SV_U
Model

Figure S8. Comparison of 5-fold test RMSE for linear regression (LR) and SVM (SV) models with no feature selection versus models with feature selection using the intersection (I) and union (U) sets for different fingerprint/property combinations. (a) ECFP2/ρ. (b) ECFP10/ρ. (c) ECFP2/E. (d) ECFP10/E. (e) ECFP2/T_g. (f) ECFP10/T_g. (g) ECFP2/T_m. (h) ECFP10/T_m.

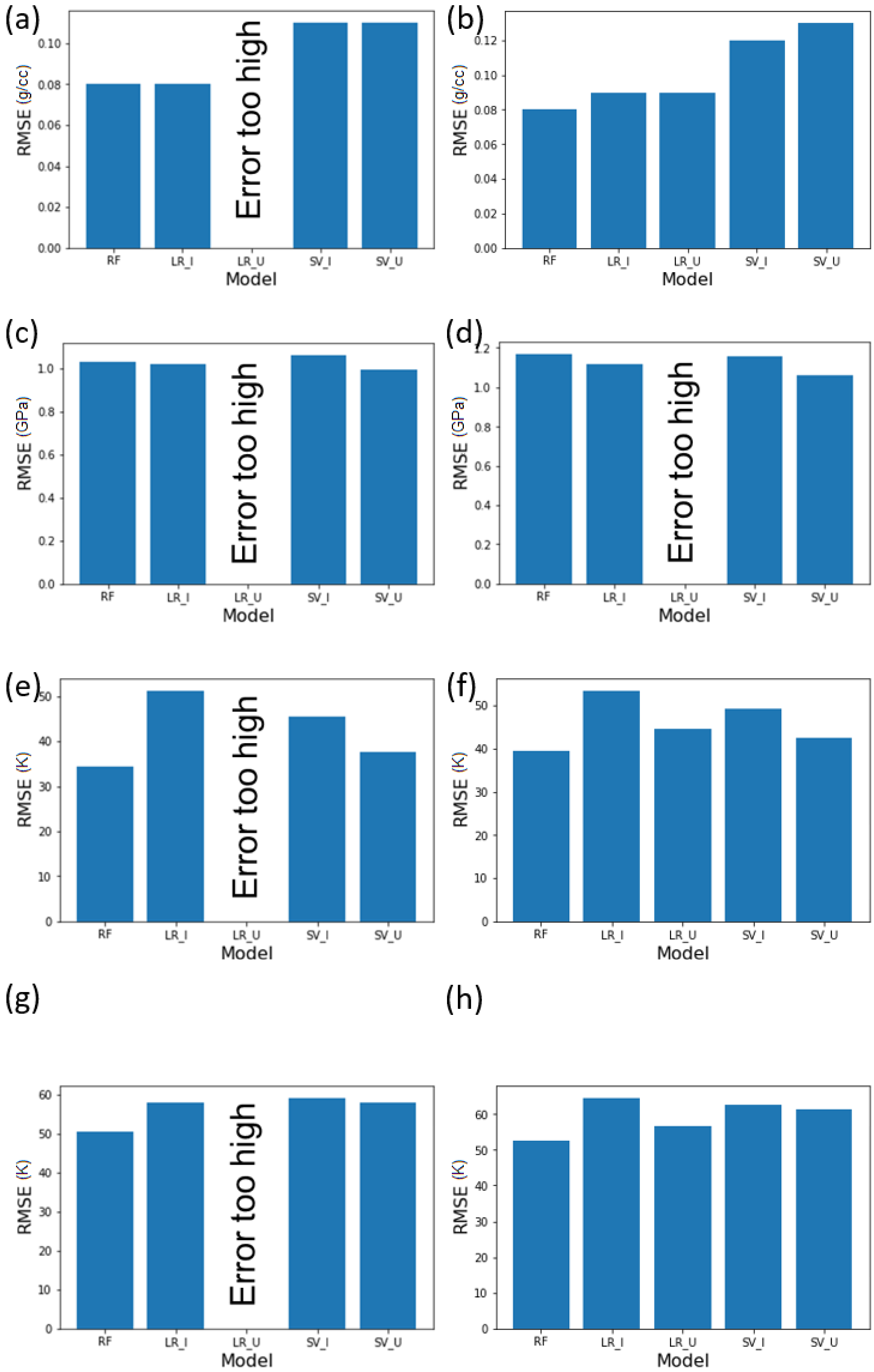

(a)
RMSE (g/cc)
0.00
0.02
0.04
0.06
0.08
0.10
Error too high
RF
LR_I
LR_U
SV_I
SV_U
Model
(b)
RMSE (g/cc)
0.00
0.02
0.04
0.06
0.08
0.10
0.12
RF
LR_I
LR_U
SV_I
SV_U
Model
(c)
RMSE (GPa)
0.0
0.2
0.4
0.6
0.8
1.0
Error too high
RF
LR_I
LR_U
SV_I
SV_U
Model
(d)
RMSE (GPa)
0.0
0.2
0.4
0.6
0.8
1.0
1.2
Error too high
RF
LR_I
LR_U
SV_I
SV_U
Model
(e)
RMSE (K)
0
10
20
30
40
50
Error too high
RF
LR_I
LR_U
SV_I
SV_U
Model
(f)
RMSE (K)
0
10
20
30
40
50
RF
LR_I
LR_U
SV_I
SV_U
Model
(g)
RMSE (K)
0
10
20
30
40
50
60
Error too high
RF
LR_I
LR_U
SV_I
SV_U
Model
(h)
RMSE (K)
0
10
20
30
40
50
60
RF
LR_I
LR_U
SV_I
SV_U
Model

Figure S9. Comparison of 5-fold test RMSE for random forest (RF) models versus for linear regression (LR) and SVM (SV) models with feature selection using the intersection (I) and union (U) sets for different fingerprint/property combinations. (a) ECFP2/ρ. (b) ECFP10/ρ. (c) ECFP2/E. (d) ECFP10/E. (e) ECFP2/T_g. (f) ECFP10/T_g. (g) ECFP2/T_m. (h) ECFP10/T_m.

QSPR feature sets

The QSPR descriptors used in this work were:

1. Number of heavy atoms
2. Number of hydrogen bonding groups (amide, urea, urethane, hydroxyl, carboxylic acid, sulfonic acid)
3. Number of rotational degrees of freedom (defined in Bicerano) [3]
4. Number of fused rings (excluding spiro and bridged rings; defined in Bicerano) [3]
5. Number of aromatic rings
6. Number of rotatable bonds
7. Number of imide groups
8. Number of amide groups
9. Number of urea groups
10. Number of urethane groups
11. Number of ketone groups
12. Number of methyl groups attached to aromatic atoms in the backbone

These descriptors were calculated using RDKit [4], whether directly as a counter (e.g. for aromatic rings) or as an enabler for a self-coded counter (e.g. for Bicerano's definition of rotational degrees of freedom).

Table S4 summarizes the results for all representation, model, and property combinations studied in this work.

Table S4. Summary of the best R^2 and $RMSE$ metrics for all representation/model/property combinations studied in this work.

		T_g (°C)		T_m (°C)		ρ (g/cc)		E (GPa)	
**Representation**	**Model**	R²	RMSE	R²	RMSE	R²	RMSE	R²	RMSE
ECFP2	Linear with FS	0.73	51.2	0.56	57.8	0.63	0.08	0.33	1.02
	SVM with FS	0.85	37.6	0.55	58.0	0.35	0.11	0.38	0.99
	RF	0.88	34.4	0.66	50.6	0.69	0.08	0.30	1.03
	LASSO Linear	0.87	35.5	0.66	50.4	n/a	0.14	n/a	1.21
	Ridge Linear	0.88	33.5	0.70	48.0	0.72	0.07	0.38	0.98
ECFP10	Linear with FS	0.79	44.7	0.57	56.8	0.58	0.09	0.09	1.12
	SVM with FS	0.81	42.4	0.50	61.4	0.23	0.12	0.28	1.06
	RF	0.84	39.5	0.63	52.5	0.67	0.08	0.15	1.17
	LASSO Linear	0.85	37.6	0.65	51.0	n/a	0.14	n/a	1.24

	Ridge Linear	0.84	39.3	0.68	49.0	0.77	0.07	0.28	1.04
QSPR unnormalized	Linear	0.71	53.3	0.37	68.9	0.59	0.09	0.16	1.12
	SVM	0.80	44.2	0.42	66.0	0.47	0.10	0.22	1.10
	RF	0.81	42.5	0.38	68.8	0.65	0.08	n/a	1.20
QSPR normalized	Linear	0.74	49.9	0.41	66.8	0.69	0.08	0.18	1.14
	SVM	0.79	44.4	0.45	64.4	0.44	0.10	0.21	1.11
	RF	0.83	41.0	0.42	65.9	0.66	0.08	n/a	1.28
QSPR unnormalized with CI	Linear	0.71	52.5	0.39	68.2	0.63	0.08	0.23	1.12
	SVM	0.80	44.1	0.41	66.6	0.44	0.10	0.18	1.11
	RF	0.83	40.0	0.42	66.4	0.67	0.08	0.19	1.15
QSPR normalized with CI	Linear	0.75	48.6	0.40	67.2	0.72	0.07	0.22	1.11
	SVM	0.80	43.9	0.44	65.1	0.48	0.10	0.09	1.21
	RF	0.82	41.3	0.38	68.2	0.72	0.07	n/a	1.28

References

1. Weininger, D. SMILES, a chemical language and information system. 1. Introduction to methodology and encoding rules. *J. Chem. Inform. Comput. Sci.* **1988**, *28*, 31-36.

2. Pedregosa, F.; Varoquaux, G.; Gramfort, A.; Michel, V.; Thirion, B.; Grisel, O.; Blondel, M.; Prettenhofer, P.; Weiss, R.; Dubourg, V.; Vanderplas, J.; Passos, A.; Cournapeau, D.; Brucher, M.; Perrot, M.; Duchesnay, E. Scikit-learn: Machine Learning in Python. *J. Mach. Learn. Res.* **2011**, *12*, 2825-2830.

3. Bicerano, J. *Prediction of polymer properties*, 3rd ed.; CRC Press: Boca Raton, Florida, United States, 2002; pp. 18-125.

4. Landrum, G. RDKit: Open-source cheminformatics. Available online: https://doi.org/10.5281/zenodo.2574427 (accessed on 4 June 2019).
